# *In Vitro* Evaluation of the Antimicrobial Effectiveness and Moisture Binding Properties of Wound Dressings

**DOI:** 10.3390/ijms11082864

**Published:** 2010-08-03

**Authors:** Pornanong Aramwit, Pornprom Muangman, Nantaporn Namviriyachote, Teerapol Srichana

**Affiliations:** 1 Department of Pharmacy Practice, Faculty of Pharmaceutical Sciences, Chulalongkorn University, Bangkok 10330, Thailand; E-Mail: nantaporn.nam@gmail.com; 2 Burn Unit, Trauma Division, Department of Surgery, Faculty of Medicine Siriraj Hospital, Mahidol University, Bangkok 10700, Thailand; E-Mail: pmuangman@yahoo.com; 3 Department of Pharmaceutical Technology and Drug Delivery System Excellence Center, Faculty of Pharmaceutical Sciences, Prince of Songkla University, Hat Yai, Songkla 90110, Thailand; E-Mail: teerapol.s@psu.ac.th

**Keywords:** antimicrobial, moisture absorption, wound dressing

## Abstract

A variety of silver-coated dressings and some impregnated with other chemicals are now available in the market; however, there have been few studies analyzing their comparative efficacies as antimicrobial agents. Moreover, their properties for retaining an appropriate level of moisture that is critical for effective wound healing have never been reported. Five commercially available silver-containing and chlorhexidine dressings, Urgotul SSD^®^, Bactigras^®^, Acticoat^®^, Askina Calgitrol Ag^®^ and Aquacel Ag^®^, were tested to determine their comparative antimicrobial effectiveness *in vitro* against five common wound pathogens, namely methicillin-sensitive and -resistant *Staphylococcus aureus*, *Bacillus subtilis*, *Escherichia coli* and *Pseudomonas aeruginosa*. Mepitel^®^, a flexible polyamide net coated with soft silicone, was used as a control. The zones of inhibition and both the rapidity and the extent of killing of these pathogens were evaluated. All five antimicrobial dressings investigated exerted some bactericidal activity, particularly against *E. coli*. The spectrum and rapidity of action ranged widely for the different dressings. Acticoat^®^ had a broad spectrum of action against both Gram-positive and -negative bacteria. Other dressings demonstrated a narrower range of bactericidal activities. Regarding the absorption and release of moisture, Askina Calgitrol Ag^®^ absorbed and released the most moisture from the environment. Aquacel Ag^®^ also exhibited good moisture absorption and moisture release, but to a lower degree. The other tested dressings absorbed or released very little moisture. Askina Calgitrol Ag^®^ and Aquacel Ag^®^ are good alternative dressings for treating wounds with high exudates and pus. An understanding of the characteristics of these dressings will be useful for utilizing them for specific requirements under specified conditions.

## 1. Introduction

The skin is the largest human organ and acts as an extremely effective biological barrier. Cutaneous wounds are normally open to the environment and wound beds are favorable environments for bacterial growth. Burn wounds are open wounds and present a critical threat to burns victims, especially those with large areas of burns. This is the primary reason for dehydration, systemic infection and other complications suffered by burns victims [1–3]. One key factor for the effective treatment of burns patients is to close the wound as soon as possible [4,5]. Infections, either by bacteria or fungi, can lead to deterioration of the wound healing process [6] and severe systemic complications. The use of antibacterial agents locally and/or systemically can contribute to wound healing, especially for burn wounds. Their use inhibits microbial growth on or around the wounds and provides a suitable microenvironment for healing [1,2,4].

Wound management can be facilitated with dressings designed both to act as a temporary barrier and to promote wound healing [7]. Several topical antimicrobial agents are widely applied to wound dressings. Silver, in particular, has been a preferred additive to medicated wound dressings because it has broad antimicrobial and antifungal activity [8]. However, there are various forms and formulations of silver dressings available on the market, and little is known about their comparative effectiveness as antimicrobial agents and the spectrum of microbial killing that each provides. Other antimicrobials used in dressings are chlorhexidine, polyhexamethylene biguanide (PHMB) and iodine, but none of these have provided any evidence for promoting resistance. Silver has the advantage of having broad antimicrobial activities against Gram-negative and Gram-positive bacteria and there is also minimal development of bacterial resistance. However, there have been several reports that silver-impregnated dressings used on burns patients can induce hepatotoxicity and argyria-like symptoms [9,10]. It might soon be possible to meet the specific requirements for any particular circumstance, such as dressings that can adequately inhibit microbial growth, yet exhibit minimal silver toxicity and still enhance wound healing. Moreover, an appropriate dressing should be applied to a sensitive patient with wounds infected by pathogens.

Besides antimicrobial properties, the ability to absorb moisture is also an important factor for healing. Bolton *et al*. suggested that the use of more moisture-retentive dressings generally supports faster healing compared with less moisture-retentive dressings [11]. However, comparisons of moisture absorption by dressings have never been studied even though the presence of moisture is known to accelerate the healing response compared with wounds that have been allowed to dry [12].

The objective of this study is to evaluate the antimicrobial effectiveness of five commercially available antimicrobial dressings *in vitro*. The moisture penetration of each dressing will also be investigated.

## 2. Results and Discussion

Table 1 shows the compositions of the studied coated dressings. All dressings contain either silver or chlorhexidine as an antimicrobial agent except for Mepitel^®^ (which has been used as a control).

### 2.1. Corrected Zone of Inhibition Test

The result for the zones of inhibition generated by antimicrobial agents and dressings are presented in Table 2. The data show that all products generated an inhibitory zone against most individual microorganisms. All dressings inhibited bacterial populations to some extent except Bactigras^®^, which had no activity against *P. aeruginosa*. Acticoat^®^ and Askina Calgitrol Ag^®^ produced the largest zones of inhibition, which may due to the high concentration of silvercontained in these dressings (105 mg/100 cm^2^ and 141 mg/100 cm^2^, respectively) compared with 3.75% of silver sulfadiazine in Urgotul SSD^®^ and 0.5% chlorhexidine in Bactigras^®^. It is clear that different organisms produced differently sized zones of inhibition against the same dressing. Methicillin resistant *Staphylococcus aureus* (MRSA) and *B. subtilis* were less sensitive to the tested antimicrobial dressings, as shown by a smaller zone of inhibition compared with other organisms.

Infection is a significant cause of delayed or prolonged wound healing, and high bacteria levels interfere with the progression of wound healing [16,17]. The broad antibacterial properties of silver and its derivatives have made it a good candidate and practical choice for creating silver dressings for wound care [13,14]. The results of this study show that all tested dressings investigated exerted some bactericidal activity, particularly on *E. coli*.

### 2.2. Bactericidal Activities of Antimicrobial Dressings

**Figure 1:**
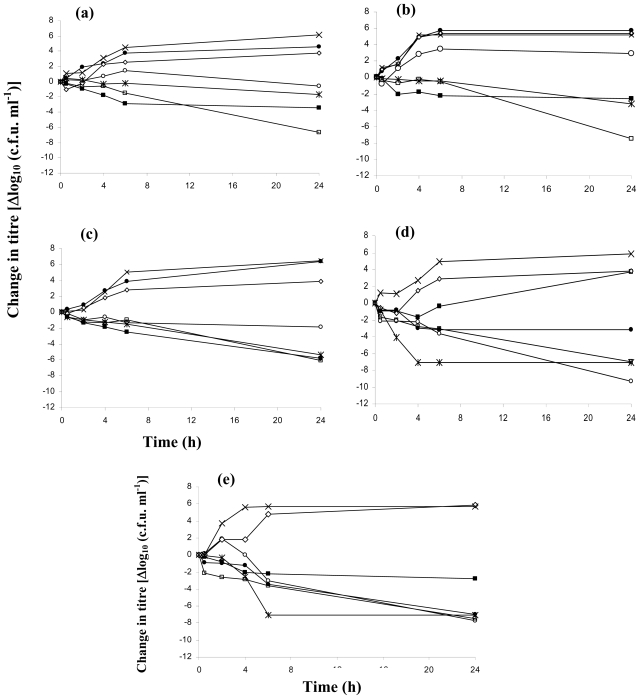
The bactericidal activities of the antimicrobial dressings against five microorganisms. Values are the means of three experiments performed in triplicate. Δlog_10_ c.f.u. ml^−1^ is the difference in Δlog_10_ c.f.u. ml^−1^ at the time of bacterial inoculation, starting from *t* = 0. Strains: (**a**) Methicillin-sensitive *Staphylococcus aureus* (ATCC 6338P); (**b**) Methicillin-resistance *Staphylococcus aureus* (ATCC 25923); (**c**) *Bacillus subtilis* (ATCC 6633); (**d**) *Pseudomonas aeruginosa* (ATCC 27853); (**e**) *Escherichia coli* (ATCC 25922) and □ represents Acticoat^®^; ○ represents Aquacel Ag^®^; * represents Askina Calgitrol Ag^®^; ■ represents Bactigras^®^; • represents Urgotul SSD^®^; ⋄ represents Mepitel^®^ and × represents growth control.

The spectrum and onset of action ranged widely for the various dressings. The bactericidal activities of the antimicrobial dressings against the five microorganisms are shown in Figure 1. Bactericidal activity was indicated by a reduction in bacterial counts presented as log_10_c.f.u. (colony forming units) mL^−1^ over time. These curves also indicated the rate of bacterial killing and provided an additional index of efficacy against the described isolate [18]. The normal growth rate of each organism was represented by the growth control and that of the Mepitel^®^ dressing, which contained no antimicrobials. Overall, Acticoat^®^ seemed to be the most effective dressing against these five tested organisms, especially with Gram-positive bacteria, whereas Urgotul SSD^®^ and Bactigras^®^ seemed to have a lower antimicrobial effect compared with the other dressings. For the Gram-positive bacteria, *S. aureus* (Figure 1a,b) and *B. subtilis* (Figure 1c), the Acticoat^®^ dressing exerted maximal bactericidal activity, achieving more than a 4 log reduction of bacterial growth after 24 h. The killing patterns of *S. aureus* and *B. subtilis* by silver dressings were similar to MRSA, except for Aquacel Ag^®^, which slightly reduced both *S. aureus* and *B. subtilis* counts but had no effect on MRSA. With *P. aeruginosa* (Figure 1d), Acticoat^®^, Askina Calgitrol Ag^®^ and Aquacel Ag^®^ exhibited a good bactericidal effect. The maximal killing of *P. aeruginosa* was achieved at 4 h with Askina Calgitrol Ag^®^ and the reduction in bacterial counts was sustained. The killing pattern for *E. coli* (Figure 1e) by Askina Calgitrol Ag^®^ was similar to that for *P. aeruginosa* except for the maximal killing, which was found at 6 h. All dressings exhibited bactericidal activity and achieved more than a 4 log reduction of *E. coli* (Figure 1e) except for Bactigras^®^, which had a less pronounced effect.

Acticoat^®^ was effective and showed a broad spectrum of bactericidal activities on the bacteria tested with a long duration of action; these results are similar to those reported by Castellano *et al*. [19]. On the other hand, Askina Calgitrol Ag^®^, which contains the highest silver concentration compared with all tested dressings [14], had the most equivalent efficacy to Acticoat^®^. Since the silver form in Acticoat^®^ is nanocrystalline, the molecular size and concentration of silver are higher than those in other dressings [20,21]. Because of its nanocrystalline form, Acticoat^®^ also exhibits sustained-release of silver molecule resulting in longer duration of action. Urgotul SSD^®^ showed bactericidal effects only with Gram-negative bacteria, similar to the results reported by Ip *et al*. [18]. The antimicrobial activity of Urgotul SSD^®^ only generated from silver sulfadiazine, which shows the main activity on Gram-negative bacteria. Most dressings showed their bactericidal activity after the first hour of testing and the activity generally lasted for at least 24 h. One advantage of this rapid antibacterial action is that it allows wound healing to proceed without bacterial interference and reduces the likelihood that resistance will develop [18]. Since some dressings did not show their maximal bactericidal activity after 24 h, the limitation of this study is that it should have been extended for longer than 24 h, since some of the dressings might have a sustained effect for many days. Our study confirmed the effectiveness of silver and chlorhexidine dressings against a broad range of bacterial pathogens. With the enhanced bacterial killing effects, clinicians should be concerned that a too high level of silver could be delivered into the tissues and cause an adverse effect such as keratinocytes and fibroblasts toxicity that might affect on the recovery of wounds [18,22]. Suitable dressings for each use should be considered from the patient’s sensitivity and the possible side effects from the level of silver in the dressings.

### 2.3. Wound Dressing Water Vapor Absorption

**Figure 2:**
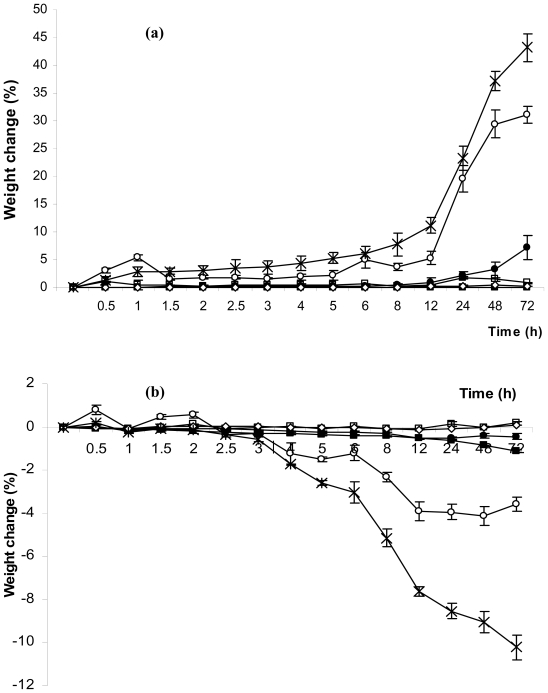
The percentage weight change of each dressing after placing into the desiccators with relative humidity at (**a**) 96.1% and (**b**) 22.4% for 0.5 to 72 h. □ represents Acticoat^®^; ○ represents Aquacel Ag^®^; * represents Askina Calgitrol Ag^®^; ■ represents Bactigras^®^; • represents Urgotul SSD^®^ and ⋄ represents Mepitel^®^.

At steady state, potassium sulfate and potassium acetate in desiccators provided a relative humidity of 96.1 ± 1.5% and 22.4 ± 1.3% at 30 °C, respectively. Figure 2 shows the percentage weight change of each dressing after being placed into the desiccators at a relative humidity of 96.1% (Figure 2a) and 22.4% (Figure 2b), respectively for 0.5 to 72 h. Acticoat^®^, Bactigras^®^, Mepitel^®^ and Urgotul SSD^®^ absorbed or released very little moisture from the dressing at any humidity, whereas Askina Calgitrol Ag^®^ absorbed and released the most moisture in humid conditions. After being placed in high humidity, Askina Calgitrol Ag^®^ started to absorb moisture within 30 min and showed a significant weight change after 12 h. It also absorbed moisture close to 50% of its initial weight after being placed in a high humidity environment for 72 h and still did not reach a saturated condition. Nevertheless, it started to release moisture after placing it in a low humidity environment for 3 h and released approximately 10% of its weight after 72 h. In addition to Askina Calgitrol Ag^®^, Aquacel Ag^®^ also showed a good absorption of moisture and had moisture release properties but to lower degree. Aquacel Ag^®^ absorbed moisture up to 30% of its weight after 72 h in a high humidity environment after which it started to reach its saturated condition, whereas it showed approximately 4% moisture release after placing it in a low humidity environment for 72 h.

Since moisture is also an important factor for wound healing, the presence of moisture avoids the delays in healing response, which occur when wounds are allowed to dry out [12]. Excessive fluid retention at the wound surface, however, can result in poor healing and the maceration of the surrounding tissue [23]. Our results indicated that Askina Calgitrol Ag^®^ absorbed and released the most moisture of the dressings tested. This was most evident with the foam dressings since there are great variations in the location of the surface moisture [24]. The greatest percentage weight change occurred with Askina Calgitrol Ag^®^ at high humidity since the foam dressings have a tendency to expand or shrink easily. Moreover, it contains superabsorbent starch co-polymer, which also increases the absorption capacity. These data indicated that Askina Calgitrol Ag^®^ is a good alternative for treating wounds with high exudates and infection. Our result is the first report on the moisture absorption property of various antimicrobial dressings that will be beneficial for clinical application.

## 3. Experimental Section

### 3.1. Corrected Zone of Inhibition Test

The antimicrobial effect of each dressing, Urgotul SSD^®^, Bactigras^®^, Acticoat^®^, Askina Calgitrol Ag^®^, Aquacel Ag^®^ and Mepitel^®^, was tested using corrected zone of inhibition method. This test was performed according to the method reported by Gallant-Behm with some modifications [25]. Briefly, the bacterial isolates were grown in broth for 4 to 6 h, and the broth was used to inoculate Muller-Hinton agar plates to form a confluent lawn. The various wound dressings (about 1 cm^2^) were applied to the center of each lawn, and all plates were incubated for 24 h at 37 °C. The inhibition zone surrounding the tested dressing was then determined. No plate dehydration was observed around the dressings and all tests were performed in triplicate with results expressed as a mean with standard deviations.

### 3.2. Bactericidal Activities of Antimicrobial Dressings

In order to determine the onset and duration of antimicrobial activity of each dressing, bactericidal activities at different time points were determined by bacterial broth culture method which was adopted from Fraser *et al*. with some modifications [26]. Dressings (about 1 cm^2^) were prepared in an aseptic manner. Each square was placed in a sterile vial and the dressing subjected to a pretreatment with 800 μL of distilled water for 10 min (according to a previously established protocol for an absorbancy test for the volume required and duration required for pretreatment). Tryptone soy broth (2.2 mL) was then added to each vial to make up to a total volume of 3 mL.

A suspension of each organism was prepared in broth from fresh colonies after overnight incubation and the turbidity was adjusted to the 0.5 McFarland standard (~1 × 10^8^ c.f.u./mL). An aliquot (10 μL) of the bacterial suspension was added to each vial containing the dressing. Control broths with and without bacterial inoculation were also included. The vials were then incubated with agitation at 35 °C in a water bath. Aliquots of 10 μL of the bacterial broth were sampled from each vial at specific time intervals (0, 30 min and 2, 4, 6 and 24 h) and serial 10-fold dilutions for each aliquot were prepared in broth. Duplicate aliquots (25 μL) of each of the serially diluted samples were spread on plates. The plates were then incubated overnight at 35 °C and colonies counted (c.f.u./mL). The dilutions that allowed quantification (10–150 colonies) were counted and the mean counts calculated. Nine vials, containing the five antimicrobial dressings as well as the control dressing (Mepitel^®^) together with the culture and the broth controls, were included in each experiment for each organism. Plate counts were measured in triplicate and each experiment was repeated three times to obtain a mean value of c.f.u. counts.

### 3.3. Wound Dressing Water Vapor Absorption

Dressings (about 9 inch^2^) were prepared in an aseptic manner and precisely weighed. Each dressing was placed in a desiccator pre-equilibrated with salts to make the relative humidity a desired value. Potassium sulfate or potassium acetate powder was placed in a desiccator to achieve a percentage relative humidity of about 90% and 20% at 30 °C, respectively, as reported by Greenspan [27]. After 30 min, 1, 1.5, 2, 2.5, 3, 4, 5, 6, 8, 12, 24, 48 and 72 h, each dressing was taken from the desiccator using sterile forceps and again precisely weighed. The equilibrium moisture absorption was determined by the percentage weight change [28]. The experiments were performed in triplicate.

## 4. Conclusions

From five-tested antimicrobial dressings, Acticoat^®^ was the most effective dressing against the tested organisms, whereas Urgotul SSD^®^ and Bactigras^®^ showed a lower antimicrobial effect compared with other dressings. Regarding the water vapor absorption activity, Askina Calgitrol Ag^®^ absorbed and released the most moisture in humid conditions. Aquacel Ag^®^ also showed good moisture absorption and release properties but to lower degree, while the other dressings hardly absorbed or released any moisture.

## Figures and Tables

**Table 1:** Compositions of the studied coated dressings [13–15].

Dressing	Compositions of the dressing materials	Formulation compositions of the coated
Urgotul SSD^®^	Lipido-colloid dressing made of a polyester mesh impregnated with hydrocolloid particles (carboxymethylcellulose), vaseline particles and silver sulfadiazine	Silver sulfadiazine content 3.75%
Bactigras^®^	Chlorhexidine acetate 0.5% in white soft paraffin tulle dressing	Chlorhexidine acetate BP 0.5%
Acticoat^®^	Nanocrystalline silver being applied to high-density polyethylene mesh which is covered to either side of a rayon-polyester core	Nanocrystalline silver 105 mg/100 cm^2^
Askina Calgitrol Ag^®^	Silver alginate wound dressing consists of an absorbent foam sheet. one surface of which is coated with an alginate matrix containing ionic silver together with a cleanser, moisturizer and a superabsorbent starch co-polymer	Silver 141 mg/100 cm^2^
Aquacel Ag^®^	Sodium carboxymethylcellulose fibers containing 1.2% ionic silver. In the presence of exudate, the dressing absorbs liquid to form a gel, binding sodium ions and releasing silver ions	Silver 8.3 mg/100 cm^2^
Mepitel^®^	Porous, semi-transparent, low-adherent wound contact layer, consisting of a flexible polyamide net coated with soft silicone	None

**Table 2:** Corrected zone of inhibitions (mm) generated by topical antimicrobial dressings.

Microorganism	Urgotul SSD^®^	Bactigras^®^	Acticoat^®^	Askina Calgitrol Ag^®^	Aquacel Ag^®^	Mepitel^®^
*S. aureus*	1.41 ± 0.86	1.13 ± 0.42	13.30 ± 0.78	24.33 ± 3.12	12.97 ± 0.85	0.00
*MRSA*	0.19 ± 0.11	0.36 ± 0.33	6.69 ± 0.14	8.11 ± 4.33	1.84 ± 0.95	0.00
*B. subtilis*	2.39 ± 2.11	7.12 ± 1.24	10.98 ± 0.49	5.62 ± 1.48	6.69 ± 1.39	0.00
*P. aeruginosa*	9.05 ± 3.34	0	17.62 ± 4.82	21.08 ± 0.89	22.56 ± 1.77	0.00
*E. coli*	6.44 ± 1.22	0.78 ± 0.16	15.98 ± 0.84	12.42 ± 0.69	10.58 ± 0.47	0.00
